# Association between the surgical approach and prognosis of spontaneous supratentorial deep intracerebral hemorrhage

**DOI:** 10.1038/s41598-024-54639-z

**Published:** 2024-02-18

**Authors:** Hui Shi, Xingwei Tan, Yongbing Deng, Minglian He, Dongsheng Chen, Weichong Zhou, Xiaoyong Tang, Yang Liu, Min Cui

**Affiliations:** 1 Department of Neurosurgery, Yongchuan Hospital of Chongqing Medical University, Chongqing, 402160 China; 2 Department of Neurosurgery, Chongqing Emergency Medical Center, Chongqing University Central Hospital, 1 Jiankang Road, Yuzhong District, Chongqing, 400010 China; 3 Department of Neurology, The Third Affiliated Hospital of Chongqing Medical University, Chongqing, 401120 China; 4 Chongqing Key Laboratory of Emergency Medicine, Chongqing Emergency Medical Center, Chongqing University Central Hospital, Chongqing, 400010 China; 5 Institute of Hepatopancreatobiliary Surgery, Southwest Hospital, Army Medical University (Third Military Medical University), Chongqing, 400038 China

**Keywords:** Neuroscience, Health care, Medical research, Neurology

## Abstract

The association between surgical approach and prognosis in patients with spontaneous supratentorial deep intracerebral hemorrhage is unclear. We aimed to explore the association between surgical approach and prognosis in these patients. A retrospective cohort of 311 patients from 3 centers who were treated with surgery 24 h after ictus was recruited. The surgical procedure involved removing the intracerebral hematoma using an aspirator through either the cortical approach or Sylvian fissure approach, assisted by an endoscope or microscope. The primary outcome was the one-year modified Rankin scale (mRS) score. The association between the surgical approach and the one-year mRS score was explored by using ordinal logistic regression and binary logistic regression. Baseline characteristics were balanced by propensity score matching and inverse propensity score weighting. In the adjusted analysis, compared with the cortex approach group, the Sylvian fissure approach group had better one-year mRS scores when analyzed as an ordinal variable (3.00 [2.00–4.00] vs. 4.00 [3.00–5.00]; adjusted odds ratio, 3.15; 95% CI, 1.78–5.58; *p* < 0.001) and a dichotomous variable (74.14% vs. 49.01%; adjusted odds ratio, 6.61; 95% CI, 2.75–15.88; *p* < 0.001). Surgical approach was not significantly associated with rebleeding (*p* = 0.88) or three-month mortality (*p* = 0.81). In univariate analysis after propensity score matching, there were significant differences in one-year mRS score between the two groups (*p* < 0.001), and there were no significant differences in rebleeding (Fisher’s exact test, *p* > 0.999) or three-month mortality (Fisher's exact test, *p* > 0.999). Inverse probability weighted regression analysis showed better one-year mRS scores when analyzed as an ordinal variable (adjusted odds ratio, 3.03; 95% CI, 2.17–4.17; *p* < 0.001) and a dichotomous variable (adjusted odds ratio, 3.11; 95% CI, 2.16–4.77; *p* < 0.001) in the Sylvian fissure approach group; the surgical approach was not significantly associated with rebleeding (*p* = 0.50) or three-month mortality (*p* = 0.60). In the surgical treatment of patients with spontaneous supratentorial deep intracerebral hemorrhage, the Sylvian fissure approach may lead to a better functional outcome compared with the cortex approach. Future prospective studies are warranted to confirm this finding.

## Introduction

Spontaneous intracerebral hemorrhage is nontraumatic intracranial bleeding that occurs in 6.5–19.6% of patients with acute stroke, has high rates of disability and fatality, and a poor prognosis^1,2^. In recent years, surgery has been increasingly performed for the treatment of spontaneous intracerebral hemorrhage. Although prospective studies, including the Surgical Trial in Intracerebral Hemorrhage (STICH) and Minimally Invasive Surgery plus rtPA for Intracerebral Hemorrhage Evacuation (MISTIE) ^3–7^, did not directly confirm that surgery improved functional outcomes in patients with spontaneous intracerebral hemorrhage, subgroup analyses of patients in the MISTIE III trial and retrospective studies of STICH II patients^7,8^ showed that the hematoma volume was significantly reduced after surgery and patients had better neurological outcomes. Preclinical animal experimental data^9–12^ have shown that early removal of hematoma after intracerebral hemorrhage can improve mechanical compression and reduce inflammatory cell aggregation, thus improving prognosis. A prospective clinical study^13^ has indicated that early computerized tomographic image–guided endoscopic surgery for the removal of acute intracerebral hematoma may contribute to neurological recovery in patients. Therefore, further exploration of surgical treatments for patients with spontaneous intracerebral hemorrhage is necessary. For supratentorial deep intracerebral hemorrhage, it is impossible to completely avoid damaging the cortex or deep white matter fibers with the current surgical approach. Therefore, it is important to choose an appropriate surgical approach that will reduce damage to the cortex and deep white matter fibers as much as possible. The basal ganglia is the most common location of supratentorial deep intracerebral hemorrhage^14^. The Sylvian fissure approach is one of the surgical approaches for basal ganglia hemorrhage^15,16^. Due to the abundance of vessels in the Sylvian fissure, separation of vessels is an important obstacle to this approach, so the clinical application of this approach is poor^17^. However, if the Sylvian fissure approach is more beneficial to improving the functional outcome of patients with supratentorial deep intracerebral hemorrhage, then dissecting the vessels in the Sylvian fissure may be worthwhile. In this study, patients with spontaneous supratentorial deep intracerebral hemorrhage were the research subjects, and the cortex approach was used as the control procedure to explore the association between the Sylvian fissure approach and prognosis.

## Methods

This article follows the Strengthening the Reporting of Observational Studies in Epidemiology guidelines^18^. Data are available from the corresponding author upon reasonable request.

### Patients

This multicenter retrospective cohort study was approved by the Ethics Committees of the participating centers (the Ethics Committee of Chongqing University Central Hospital, the Ethics Committee of Yongchuan Hospital of Chongqing Medical University, and the Ethics Committee of the Third Affiliated Hospital of Chongqing Medical University) and registered in the Chinese Clinical Trial Center (registration number: ChiCTR2300069932). All methods were performed in accordance with the relevant guidelines and regulations. Patients at 3 centers (Chongqing University Central Hospital, Yongchuan Hospital of Chongqing Medical University, and the Third Affiliated Hospital of Chongqing Medical University) were enrolled between January 2017 and December 2021.

Inclusion criteria: first-time spontaneous intracerebral hemorrhage; age ≥ 18 years; hematoma located deep supratentorial; hematoma volume ≥ 20 ml; microscope-assisted or endoscope-assisted craniotomy with small bone window; surgery performed within 24 h after ictus. Exclusion criteria: hemorrhage of specific causes (aneurysm, cerebrovascular malformation, tumor, trauma); the main hematoma located in the subcortex or thalamus; the hematoma expanded into the ventricle, causing ventricle cast or hydrocephalus; the hematoma expanded into the brain stem; one or both pupils dilated and a Glasgow coma scale (GCS) score < 8; GCS score ≤ 4; the ictus time is not clear; platelet count < 100 × 10^9^; coagulation dysfunction (International Normalized Ratio > 1.4); severe hepatic insufficiency, renal insufficiency, cardiac insufficiency and pulmonary insufficiency before ictus; mRS score ≥ 3 before ictus; malignant tumor; craniectomy (patients who underwent craniectomy in the reoperation were not excluded); hematoma puncture surgery (stereotaxis, hard and soft channels); bilateral intracerebral hemorrhage.

### Treatment protocol

The treatment protocol included surgical treatments (transfrontal endoscope-assisted surgery and transtemporal endoscope-assisted or microscope-assisted surgery), nonsurgical treatments (monitoring and controlling blood pressure, hemostasis, etc.), and the management of complications during hospitalization (such as pulmonary infection, gastrointestinal bleeding, etc.). Details are available in the Supplement.

In transfrontal endoscope-assisted surgery, a small bone flap was created through a straight incision along the midline of the forehead. The hematoma’s location was determined using noncontrast computed tomography image, and the endoscopic sheath tube was inserted after transfrontal cortical puncture. The intracerebral hematoma was then removed with endoscopic assistance through an aspirator. Following hemostasis in the operative area, a drainage tube was inserted. The scalp was sutured after securing the bone flap.

In transtemporal endoscope-assisted or microscope-assisted surgery, a small bone flap was created through a straight incision in the projection of Sylvian fissure's body surface. Puncture or fistulization of the insular cortex followed Sylvian fissure separation (or occurs directly in the cortex of the superior temporal gyrus). The intracerebral hematoma was then removed with an aspirator assisted by an endoscope or microscope. Following hemostasis in the operative area, a drainage tube was inserted. The scalp was sutured after securing the bone flap.

### Imaging protocol

All patients underwent head noncontrast computed tomography (CT) examination on admission, and computed tomography angiography examination was completed on admission or before surgery to exclude aneurysms, arteriovenous malformations, etc. If the patient's arousal level before surgery was worse than that on admission, the head noncontrast CT examination was reexamined before surgery. Postoperative head noncontrast CT examination was performed (within 1 day, 1–3 days, 3–7 days) during subsequent hospitalization according to the patient's condition or at an interval of 7–10 days.

### Clinical assessments

The observational indexes included sex, age, smoking history, drinking history, underlying diseases (diabetes, hypertension), antiplatelet therapy, anticoagulant therapy, systolic blood pressure (SBP) on admission, diastolic blood pressure (DBP) on admission, preoperative arousal level, preoperative GCS score, time of ictus, start time of surgery, surgical method (microscope-assisted or endoscope-assisted), surgical approach (cortex or Sylvian fissure), hematoma location (left or right), preoperative hematoma volume, preoperative midline shift, preoperative intraventricular hematoma, postoperative residual hematoma, postoperative rebleeding, postoperative reoperation and surgical methods.

Patients were divided into the cortex approach (temporal cortex or frontal cortex) (control group) and the Sylvian fissure approach (treatment group). The primary outcome was mRS score at the one-year follow-up (one-year mRS score). The secondary outcomes were postoperative rebleeding (rebleeding) and mortality at the three-month follow-up (three-month mortality). The minimum follow-up period was 1 month, the maximum follow-up period was 12 months, and the median follow-up period was 12 months (95% confidence interval [CI]: 11.98–12.02). Patients were followed up via telephone or in outpatient clinics. The follow-up measurement was the mRS score.

### Definitions and measurements

We defined supratentorial deep intracerebral hemorrhage and surgical timing. Additionally, we described the assessment methods for pertinent indicators, including arousal level, GCS score, mRS score, hematoma volume, midline shift, the presence or absence of postoperative residual hematoma/rebleeding in the operation area, and the indications for reoperation after the first surgery. Details are available in the Supplement.

### Statistical analysis

We first evaluated the association among the clinical features, imaging features and outcomes by using a univariate analysis and the association between the surgical approach and outcomes by using an adjusted analysis. The baseline characteristics were balanced by propensity score matching and inverse probability weighting with propensity scores. Finally, the results were verified by propensity score adjustment analysis. Unordered categorical variables were expressed as counts and percentages. Ordinal categorical variables and continuous variables were expressed as medians (interquartile range [IQR]).

Univariate analyses included the chi-square test, Fisher’s exact test, Mann–Whitney U test and Kruskal–Wallis test. In addition to the surgical approach, the independent variables with a *p* value < 0.05 in the univariate analysis were included in the multivariate logistic regression and multivariate ordinal logistic regression adjusted analysis, and the independent variables were verified by the variance inflation factor (VIF) to have no collinearity. The ordinal logistic regression model passed the test of parallel lines. 1-year mRS score was analyzed as an ordinal variable and a dichotomous variable.

#### Propensity score

Univariate analysis was performed on the clinical and imaging features of the two groups of patients to determine the matching variables. The two groups were propensity score matched 1:1. The nearest neighbor matching method was used, and the caliper value was 0.2 of the standard deviation of the propensity score. The matching effect was evaluated by standard mean difference (SMD). After propensity score matching, the chi-square test and Mann‒Whitney U test were used to compare the outcomes between the two groups. The association between surgical approach and outcomes was evaluated by logistic regression after inverse probability weighting with propensity scores.

#### Sensitivity analysis

In the multivariate logistic regression model, the robustness of the results was assessed by propensity score adjustment analysis.

SPSS version 25.0 (IBM) and R version 4.2.2 (R Foundation for Statistical Computing) were used for statistical analysis. Propensity score matching was performed using the MatchIt package (version 4.5.0). All tests were two-sided, and a *P* value < 0.05 was considered statistically significant.

### Ethical approval

Our institutional review boards (the Ethics Committee of Chongqing University Central Hospital, the Ethics Committee of Yongchuan Hospital of Chongqing Medical University, and the Ethics Committee of the Third Affiliated Hospital of Chongqing Medical University) approved this retrospective study and waived the requirement for informed consent from the patients.

## Results

A total of 311 patients were included in the study cohort (Fig. S1). Of these, 217 (69.77%) were males and 94 (30.23%) were females, with a mean age of 55.00 (48.00–66.00) years. Ninety (28.94%) had a history of smoking, 96 (30.87%) had a history of drinking, 192 (61.74%) had a history of hypertension, and 19 (6.11%) had a history of diabetes. Five (1.61%) patients used antiplatelet drugs. None of the enrolled patients had a history of anticoagulant use. The systolic blood pressure on admission was 177.00 (160.00–191.00) mmHg, and the diastolic blood pressure on admission was 102.00 (94.00–115.00) mmHg. The preoperative arousal level was 3.00 (3.00–3.00). The preoperative arousal level was good in 244 (78.46%) patients, and the preoperative arousal level was bad in 67 (21.54%) patients. The preoperative GCS score was 9.00 (8.00–11.00). Microscope-assisted surgery was performed on 173 (55.63%) patients, and endoscope-assisted surgery was performed on 138 (44.37%) patients. The cortical and Sylvian fissure approaches were performed on 253 (81.35%) and 58 (18.65%) patients, respectively. There were 171 (54.98%) hematomas on the left side and 140 (45.02%) hematomas on the right side. The preoperative hematoma volume was 35.27 (27.07–46.00) ml. The preoperative midline shift was 5.70 (4.20–7.70) mm. A total of 126 (40.51%) patients had intraventricular hematomas before surgery. The surgical timing was 447.00 (326.00–731.00) min. Postoperative rebleeding occurred in 30 (9.65%) patients. Eight (2.57%) patients had residual hematomas after surgery. Eleven (3.54%) patients needed reoperation for postoperative rebleeding, of which 2 (0.64%) patients refused reoperation, and 9 (2.89%) patients underwent reoperation (4 cases of hematoma evacuation, 5 cases of hematoma evacuation and craniectomy) (Table 1).

**Table 1:** Descriptive data.

Characteristics	N (%)/Median (IQR)
No. of patients	311
Sex	
Male	217 (69.77)
Female	94 (30.23)
Age, years	55.00 (48.00–66.00)
Smoking	90 (28.94)
Drinking	96 (30.87)
Hypertension	192 (61.74)
Diabetes	19 (6.11)
Antiplatelet	5 (1.61)
Anticoagulant	0 (0)
SBP on admission, mmHg	177.00 (160.00–191.00)
DBP on admission, mmHg	102.00 (94.00–115.00)
Arousal level (ordinal variable)	3.00 (3.00–3.00)
Arousal level (dichotomous variable)	
Good^a^	244 (78.46)
Bad^b^	67 (21.54)
GCS score	9.00 (8.00–11.00)
Surgical method	
Microscope	173 (55.63)
Endoscope	138 (44.37)
Surgical approach	
Cortex	253 (81.35)
Sylvian fissure	58 (18.65)
Hematoma location	
Right	140 (45.02)
Left	171 (54.98)
Hematoma volume, ml	35.27 (27.07–46.00)
Midline shift, mm	5.70 (4.20–7.70)
Intraventricular hematoma	126 (40.51)
Surgical timing, min	447.00 (326.00–731.00)
Rebleeding	30 (9.65)
Residual hematoma	8 (2.57)
Reoperation	9 (2.89)
One-year mRS score (ordinal variable)	3.00 (3.00–5.00)
One-year mRS score (dichotomous variable)	
Good^c^	167 (53.70)
Bad^d^	144 (46.30)
Three-month mortality	23 (7.40)

IQR, interquartile range; SBP, systolic blood pressure; DBP, diastolic blood pressure; GCS, Glasgow Coma Scale; mRS, modified Rankin scale.

^a^Arousal level = 1, 2, 3.

^b^Arousal level = 4, 5.

^c^mRS score = 1, 2, 3.

^d^mRS score = 4, 5, 6.

In the univariate analysis, surgical approach was significantly associated with one-year mRS as an ordinal variable (*p* < 0.001) (Tables S1, S2) and one-year mRS as a dichotomous variable (*p* = 0.001) (Table S3). There was no significant association between surgical approach and postoperative rebleeding (*p* = 0.20) (Table S4) or three-month mortality (Table S5) (*p* = 0.59). In the adjusted analysis, the Sylvian fissure approach group had better one-year mRS scores as an ordinal variable (3.00 [2.00–4.00] vs. 4.00 [3.00–5.00]; adjusted odds ratio, 3.15; 95% CI, 1.78–5.58; *p* < 0.001) (Table 2) and as a dichotomous variable (74.14% vs. 49.01%; adjusted odds ratio, 6.61; 95% CI, 2.75–15.88; *p* < 0.001) (Table 3); the surgical approach was not significantly associated with rebleeding (*p* = 0.88) (Table S6) or three-month mortality (Table S7) (*p* = 0.81).

**Table 2:** Multivariable ordinal logistic regression of one-year mRS score (ordinal variable).

Variables	Odds ratio (95% CI)	*p* value	VIF
Sex	1.19 (0.73–1.92)	0.49	1.16
Hypertension	1.39 (0.89–2.15)	0.15	1.09
Diabetes	4.51 (1.75–11.59)	< 0.001	1.07
Arousal level	1.08 (0.58–2.04)	0.80	1.63
Surgical approach	3.15 (1.78–5.58)	< 0.001	1.12
Intraventricular hematoma	1.69 (1.09–2.62)	0.02	1.11
Rebleeding	3.89 (1.84–8.23)	< 0.001	1.08
Age, years	1.10 (1.08–1.12)	< 0.001	1.13
GCS score	1.15 (1.01–1.30)	0.03	1.66
Hematoma volume, ml	1.01 (0.99–1.02)	0.55	1.67
Midline shift, mm	1.17 (1.06–1.30)	< 0.001	1.55

The Sylvian fissure approach was the treatment group^a^, and the cortex approach was the control group^b^.

Test of parallel lines (χ^2^ = 48.20, *p* = 0.31).

mRS, modified Rankin scale; CI, confidence interval; VIF, variance inflation factor; DBP, diastolic blood pressure; GCS, Glasgow Coma Scale.

^a^One-year mRS score (3.00 [2.00–4.00]).

^b^One-year mRS score (4.00 [3.00–5.00]).

**Table 3:** Multivariable logistic regression of one-year mRS score (dichotomous variable).

Variables	Odds ratio (95% CI)	*p* value	VIF
Sex	1.37 (0.65–2.88)	0.41	1.39
Smoking	1.35 (0.65–2.82)	0.42	1.23
Hypertension	0.76 (0.41–1.41)	0.39	1.08
Arousal level	1.43 (0.56–3.68)	0.46	1.71
Surgical method	7.93 (3.43–18.33)	< 0.001	1.59
Surgical approach	6.61 (2.75–15.88)	< 0.001	1.32
Intraventricular hematoma	0.62 (0.33–1.16)	0.14	1.11
Rebleeding	0.20 (0.07–0.58)	0.003	1.08
Age, years	0.91 (0.88–0.94)	< 0.001	1.36
DBP, mmHg	1.00 (0.99–1.02)	0.72	1.19
GCS score	1.31 (1.07–1.60)	0.01	1.79
Hematoma volume, ml	0.97 (0.95–0.99)	0.03	1.79
Midline shift, mm	0.96 (0.83–1.10)	0.54	1.62
Surgical timing, min	1.00 (0.99–1.00)	0.68	1.13

The Sylvian fissure approach was the treatment group^a^, and the cortex approach was the control group^b^.

mRS, modified Rankin scale; CI, confidence interval; VIF, variance inflation factor; DBP, diastolic blood pressure; GCS, Glasgow Coma Scale.

^a^Good one-year mRS score (74.14%).

^b^Good one-year mRS score (49.01%).

### Propensity score

**Figure 1:**
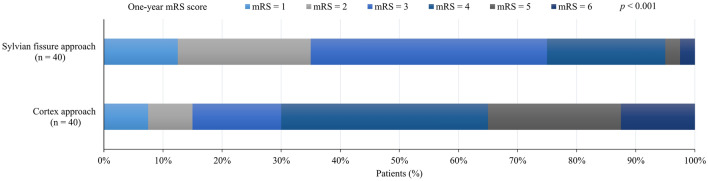
Distribution of one-year modified Rankin scale (mRS) score (ordinal variable) according to type of surgical approach in the propensity score-matched cohort. The scores in the two groups were significantly different (Mann‒Whitney U test [Z = − 3.83, *p* < 0.001]). The modified Rankin scale score ranges from 1 to 6, with higher scores indicating worse outcomes.

The univariate analysis of clinical characteristics and image characteristics of the two groups is shown in Table S8, and the matching effect is shown in Table 4. After propensity score matching, the Sylvian fissure approach group had better one-year mRS score (Mann–Whitney U test, Z = − 3.83, *p* < 0.001 [Fig. 1]; chi-square test, χ^2^ = 16.24, *p* < 0.001); there were no significant differences in rebleeding (Fisher’s exact test, *p* > 0.999) or three-month mortality between the two groups (Fisher’s exact test, *p* > 0.999). Inverse probability weighted regression analysis showed better one-year mRS scores as an ordinal variable (adjusted odds ratio, 3.03; 95% CI, 2.17–4.17; p < 0.001) and as a dichotomous variable (adjusted odds ratio, 3.11; 95% CI, 2.16–4.77; *p* < 0.001) in the Sylvian fissure approach group; the surgical approach was not significantly associated with rebleeding (adjusted odds ratio, 0.82; 95% CI, 0.45–1.48; *p* = 0.50) or three-month mortality (adjusted odds ratio, 0.84; 95% CI, 0.43–1.63; *p* = 0.60).

**Table 4:** Propensity score matching and propensity score weighting.

Variables	Original study cohort				Propensity score matching				Propensity score weighting			
	Cortex n (%)	Sylvian fissure n (%)	*p* value	SMD	Cortex n (%)	Sylvian fissure n (%)	*p* value	SMD	Cortex n (%)	Sylvian fissure n (%)	*P *value	SMD
N	253	58			40	40			309.31	243.18		
Hypertension	163 (64.43)	29 (50.00)	0.04	0.289	23 (57.50)	23 (57.50)	> 0.999	< 0.001	189.52 (61.27)	105.76 (43.49)	0.11	0.362
Surgical method				2.757			0.49	0.274			0.26	0.419
Microscope	117 (46.25)	56 (96.55)	< 0.001		40 (100)	38 (95.00)			171.33 (55.39)	182.24 (74.94)		
Endoscope	136 (53.75)	2 (3.45)			0 (0)	2 (5.00)			137.98 (44.61)	60.94 (25.06)		
Hematoma volume, median (IQR), ml	36.97 (28.18–49.17)	30.05 (22.08–36.15)	< 0.001	1.151	30.09 (22.97–41.03)	33.70 (28.28–38.09)	0.44	0.055	34.61 (26.83–46.51)	30.35 (28.02–37.17)	0.08	0.452
Midline shift, median (IQR), mm	6.00 (4.50–7.85)	4.25 (3.00–5.99)	< 0.001	0.649	5.15 (4.00–8.00)	5.35 (4.00–6.70)	0.44	0.229	5.70 (4.30–7.70)	6.00 (4.02–6.84)	0.87	0.023

SMD, standard mean difference; IQR, interquartile range.

### Sensitivity analysis

After propensity score adjustment, the Sylvian fissure approach group had better one-year mRS scores as a dichotomous variable (adjusted odds ratio, 5.83; 95% CI, 2.38–14.27; *p* < 0.001) (Table S9); the surgical approach was not significantly associated with rebleeding (*p* = 0.90) (Table S10) or three-month mortality (Table S11) (*p* = 0.48).

## Discussion

In the adjusted analysis, compared with the cortex approach group, the Sylvian fissure approach group had better one-year mRS scores as an ordinal variable (3.00 [2.00–4.00] vs. 4.00 [3.00–5.00]; adjusted odds ratio, 3.15; 95% CI, 1.78–5.58; *p* < 0.001) and as a dichotomous variable (74.14% vs. 49.01%; adjusted odds ratio, 6.61; 95% CI, 2.75–15.88; *p* < 0.001). Surgical approach was not significantly associated with rebleeding (*p* = 0.88) or three-month mortality (*p* = 0.81). In univariate analysis after propensity score matching, there were significant differences in one-year mRS score between the two groups (*p* < 0.001), and there were no significant differences in rebleeding (Fisher's exact test, *P* > 0.999) or three-month mortality (Fisher’s exact test, *P* > 0.999). Inverse probability weighted regression analysis showed better one-year mRS score as an ordinal variable (adjusted odds ratio, 3.03; 95% CI, 2.17–4.17; *p* < 0.001) and as a dichotomous variable (adjusted odds ratio, 3.11; 95% CI, 2.16–4.77; *p* < 0.001) in the Sylvian fissure approach group; the surgical approach was not significantly associated with rebleeding (*p* = 0.50) or three-month mortality (*p* = 0.60). Sensitivity analyses were consistent with the above results.

The Sylvian fissure approach for the evacuation of supratentorial deep intracerebral hemorrhage was first proposed by Suzuki^19^ in 1972, but it requires skilled dissection techniques of the vessels in the Sylvian fissure, which limits the options for less experienced surgeons^20^. Previous studies on the evacuation of spontaneous intracerebral hemorrhage through the Sylvian fissure approach are mainly case reports with small samples and short-term follow-up^21–24^, and the association between the Sylvian fissure approach and the prognosis of patients with spontaneous intracerebral hemorrhage is controversial. A total of 33 patients with spontaneous intracerebral hemorrhage were reported in the literature^25^, including 14 (44.42%) patients who underwent the Sylvian fissure approach and 19 (55.58%) patients who underwent the cortex approach. All patients had a hematoma volume ≥ 60 ml. There were no significant differences in 30-day mortality (14.29% vs. 31.58%) or Glasgow Outcome Scale (GOS) score ≥ 4 (35.71% vs. 31.58%) between the two groups. The large hematoma volume and short follow-up time of the patients included in this study may not fully reflect the advantages of the Sylvian fissure approach. A total of 66 patients with spontaneous intracerebral hemorrhage were reviewed^26^; 47 (71.2%) were treated with surgery through the Sylvian fissure approach, and 19 (28.8%) were treated with medication. The hematoma volume was ≥ 30 ml in both groups. The two groups differed significantly in mortality (34.0% vs. 63.1%) and moderate disability (6-month GOS score = 4) (27.7% vs. 5.3%). Another study^27^ reviewed 80 patients with spontaneous intracerebral hemorrhage, including 45 (56.25%) patients treated with the Sylvian fissure approach and 35 (43.75%) patients treated with the cortex approach. There was a significant difference in functional outcome (Activities of Daily Living (ADL) score ≤ 3) at 6 months after surgery between the two groups (75% vs. 50%). A meta-analysis^28^ included 659 patients from 7 studies, of which 329 (49.92%) patients were treated with the Sylvian fissure approach and 330 (50.08%) patients were treated with the cortex approach. The Sylvian fissure approach group had a higher hematoma clearance rate (odds ratio = 2.361, 95% CI: 1.443–3.861) and better postoperative functional outcome (GOS score ≥ 4 or ADL score ≤ 3) (odds ratio = 2.248, 95% CI: 1.498–3.160).

The results of this study suggest that compared with the cortex approach, the Sylvian fissure approach can obtain better one-year mRS scores without increasing the rate of postoperative rebleeding. The Sylvian fissure approach can avoid damaging the important functional cortex by cutting the insular cortex, especially in patients with dominant hemisphere hemorrhage^29–31^. The natural gap provided by the Sylvian fissure approach reduces the distance from the cortex to the hematoma^32^; during the operation, with the release of cerebrospinal fluid and the evacuation of the hematoma, the Sylvian fissure provides sufficient traction space, thereby reducing additional damage to the cortex and deep white matter fibers such as the internal capsule. These advantages of the Sylvian fissure approach may lay the foundation for a better functional outcome for patients. In a recent retrospective cohort study^33^ involving 134 consecutive patients with supratentorial intracerebral hemorrhage who underwent surgery, 66 patients underwent endoscopic hematoma evacuation under local anesthesia, while 68 patients underwent craniotomy hematoma evacuation under general anesthesia. Following the surgical concept proposed by the authors, where intentional retention of the hematoma was advocated to prevent additional damage to the brain, it was observed that the group undergoing endoscopic surgery under local anesthesia achieved better 6-month mRS scores. This study introduces a novel concept and foundation for surgery aimed at improving the prognosis of patients with supratentorial intracerebral hemorrhage. The integration of these concepts in the Sylvian fissure approach represents a promising avenue for further exploration to enhance the prognosis of patients with spontaneous supratentorial deep intracerebral hemorrhage.

### Limitations

This study has some limitations. First, in the sensitivity analysis, multivariate ordinal logistic regression analysis with propensity score adjustment was not performed because the parallel line test failed, which may have led to biased results. Second, the outcome assessment was not blinded and was performed at times by a member of the surgical team, although this was consistent throughout the study period. Third, because the study lacked randomization, unmeasured confounders may be present. Fourth, only a few patients took antiplatelets and no patients took anticoagulants in this study, so the applicability of the results to such patients is poor. Fifth, among the 327 patients who met the inclusion and exclusion criteria, 16 patients (4.89%) had missing values due to loss of follow-up and were subsequently excluded. Although the proportion of excluded patients was small, the potential for selection bias cannot be completely ruled out.

## Conclusions

In the surgical treatment of patients with spontaneous supratentorial deep intracerebral hemorrhage, the Sylvian fissure approach may provide better functional outcomes than the cortex approach. The results of this study provide a basis for improving the surgical efficacy for patients with spontaneous supratentorial deep intracerebral hemorrhage. Future prospective studies are warranted to confirm this finding.

### Supplementary Information

Supplementary Figure S1.

Supplementary Information 2.

## Data Availability

Data are available from the corresponding author upon reasonable request.
