# Supplementary figures and images for: Association between the surgical approach and prognosis of spontaneous supratentorial deep intracerebral hemorrhage
Source: Sci Rep. 2024 Feb 18;14:3994. doi: 10.1038/s41598-024-54639-z (PMC10874980; doi:10.1038/s41598-024-54639-z)

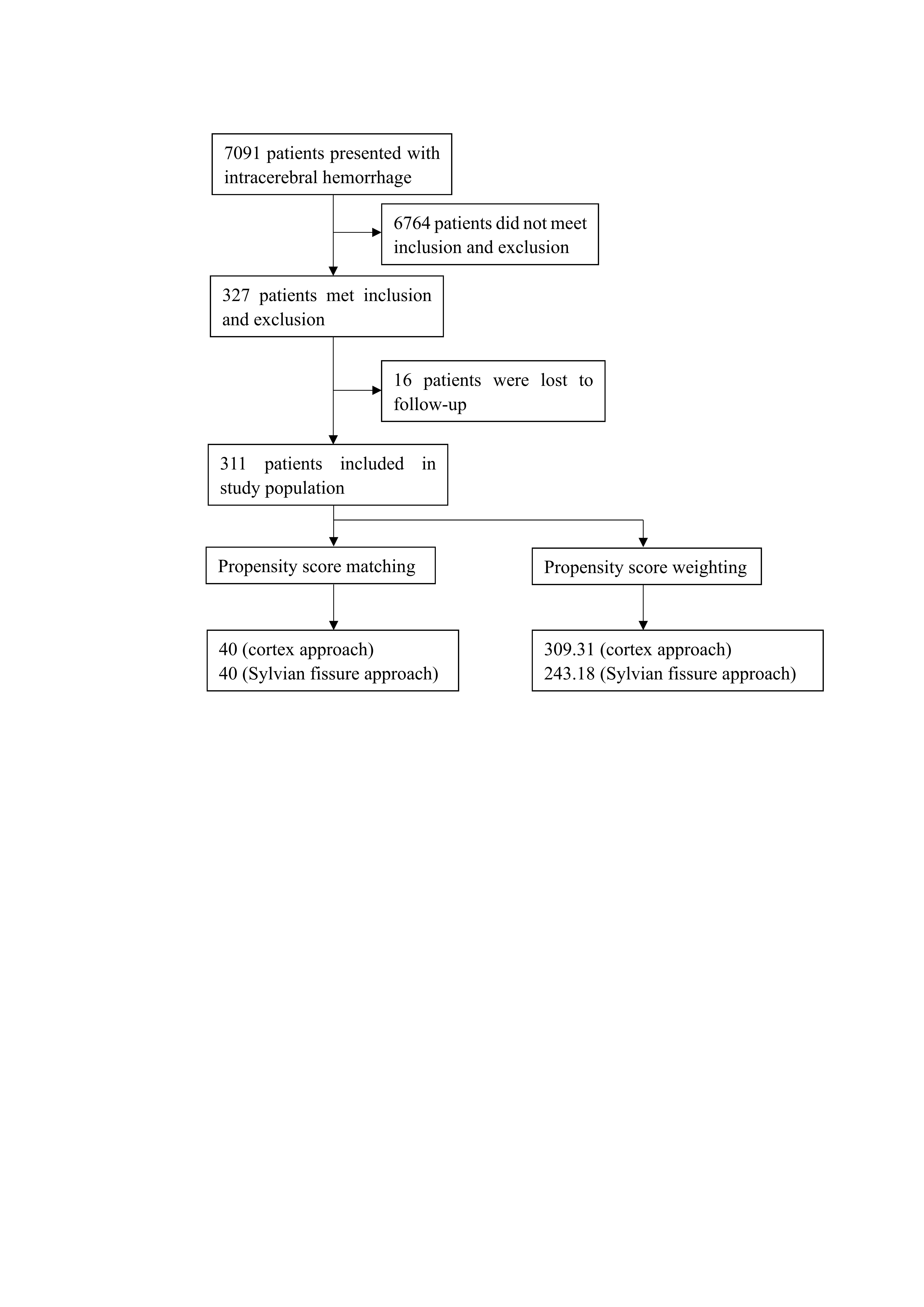

Supplement: Supplementary file 1 — Supplementary Figure S1. [file 41598_2024_54639_MOESM1_ESM.tif]
