# Supplementary material for: Association between the surgical approach and prognosis of spontaneous supratentorial deep intracerebral hemorrhage
Source: Sci Rep. 2024 Feb 18;14:3994. doi: 10.1038/s41598-024-54639-z (PMC10874980; doi:10.1038/s41598-024-54639-z)
Supplement: Supplementary file 2 — Supplementary Information 2. [file 41598_2024_54639_MOESM2_ESM.docx]

**SUPPLEMENTAL MATERIAL**

**Supplemental Methods**

**Treatment Protocol**

The surgical treatments included transfrontal endoscope-assisted surgery and transtemporal endoscope-assisted or microscope-assisted surgery. In transfrontal endoscope-assisted surgery, a 5 cm longitudinal incision was made 3 cm away from the midline of the forehead. The scalp was incised, and the skull was drilled to form a 3 cm x 4 cm bone window. The dura was cut and suspended in the bone window. The location of the hematoma was determined by using noncontrast computed tomography (CT) image, and the endoscope sheath tube was implanted after puncture through the frontal cortex. The intracerebral hematoma was removed under endoscopic assistance through an aspirator. Electrocoagulation was used when necessary. The operative area was repeatedly rinsed with normal saline and showed no active bleeding. Absorbable hemostatic gauze and a drainage tube were placed in the operative area. The dura was sutured, the bone flap was fixed with connection straps, and the scalp was sutured. The drainage tube was removed within 24 hours after surgery. In transtemporal endoscope-assisted or microscope-assisted surgery, a 5-6 cm straight incision was made at the projection of the Sylvian fissure. The scalp was incised, and the skull was drilled to form a 3 cm x 4 cm bone window. The dura was cut and suspended in the bone window. Puncture or fistulization of the insular cortex was performed after separation at the Sylvian fissure (or directly in the superior temporal gyrus cortex). The intracerebral hematoma was removed under endoscopic or microscopic assistance through an aspirator. Electrocoagulation was used when necessary. The operative area was repeatedly rinsed with normal saline and showed no active bleeding. Absorbable hemostatic gauze and a drainage tube were placed in the operative area. The dura was sutured, the bone flap was fixed with connection straps, and the scalp was sutured. The drainage tube was removed within 24 hours after surgery.

Nonsurgical treatment methods included the following: monitoring and controlling blood pressure (uradil hydrochloride, nicardipine hydrochloride, nifedipine controlled release tablets, etc.), hemostasis (caroxate sodium sulfonate injection), reducing intracranial pressure (mannitol, furosemide), atomization and dilution of sputum (acetylcysteine, ambroxol hydrochloride), turning over to prevent pressure ulcers, and barotherapy to prevent deep vein thrombosis.

In-hospital complications were managed using the following methods: for pulmonary infection, sensitive antibiotics were selected according to sputum culture (ceftazidime, piperacillin tazobactam, etc.); for gastrointestinal bleeding, drugs were selected for inhibition of gastric acid secretion and gastric mucosa protection (omeprazole, aluminum magnesium carbonate, etc.); for epilepsy, patients were given carbamazepine, sodium valproate, etc.; and for limb venous thrombosis, patients were given low-molecular-weight heparin calcium anticoagulant or underwent inferior vena cava filter placement.

**Definitions and Measurements**

Supratentorial deep intracerebral hemorrhage: most of the hematoma (≥ 50%) was in the lenticular nucleus, the head of the caudate nucleus, the clump nucleus, the internal capsule, or the external capsule. Surgical timing: the time of ictus was provided by the witness accompanying the patient; otherwise, the time of ictus was the time of the last normal state. The start time of surgery was recorded at the beginning of skin incision after anesthesia. The interval between the time of ictus and the start of surgery was defined as the surgical timing. Neurological assessment: arousal level was graded as level 1 (clear consciousness), level 2 (somnolence), level 3 (sopor), level 4 (mild coma), or level 5 (moderate coma). Grades 1-3 were considered to indicate a good level of arousal, and grades 4-5 were considered to indicate a bad level of arousal. For the GCS score, refer to reference 1. For the mRS^2-4^ score, a score of 0-3 was defined as a good outcome, and a score of 4-6 was defined as a bad outcome. The hematoma volumes were calculated by using the following method: according to head noncontrast CT (layer thickness: 0.5 cm, layer spacing: 0.5 cm), the Tada formula was applied: V (hematoma volume) = a × b × c × 1/2, where a is the longest diameter of the hematoma at the level of the maximum hematoma area, b is the longest diameter perpendicular to the longest diameter at the level of the maximum hematoma area, and c is the number of layers of the hematoma on noncontrast CT scan. The midline shift was measured by using the following methods^5^: the anterior point of the midline: frontal crest or the point of intersection between the cerebral falx and the frontal bone; the posterior point of the midline: the central point of the sagittal sinus in the parieto-occipital region; midline shift: the vertical distance from the midline to the farthest point of the septum pellucidum, the vertical distance from the midline to the third ventricle or the vertical distance from the midline to the pineal calcification. The presence or absence of postoperative residual hematoma/rebleeding in the operation area^6-9^ was determined by head noncontrast CT examination within 24 hours after surgery and intraoperative observation of the operation area. In patients whose noncontrast CT reexamination within 24 hours after surgery showed hematoma, if the shape and density of the postoperative hematoma were the same as those before surgery, there were no surgical traces (such as gas) around the postoperative hematoma, and the postoperative hematoma was in the periphery (relative to the preoperative hematoma), the hematoma was defined as a residual hematoma. Otherwise, postoperative hematoma was defined as rebleeding. A residual hematoma or rebleeding with a volume > 15 ml was included in the statistical analysis. The indications for reoperation after the first surgery were as follows: the postoperative hematoma volume was greater than or equal to the preoperative volume; the postoperative hematoma volume was reduced when compared with that before the operation but still ≥ 20 ml, and the postoperative GCS score was lower than that before the operation or the arousal level was worse than that before the operation.

**References**

**1**. Teasdale G, Jennett B. Assessment of coma and impaired consciousness. A practical scale. Lancet. 1974;2(7872):81-84. doi:10.1016/s0140-6736(74)91639-0.

**2**. van Swieten JC, Koudstaal PJ, Visser MC, Schouten HJ, van Gijn J. Interobserver agreement for the assessment of handicap in stroke patients. Stroke. 1988;19(5):604-607. doi:10.1161/01.str.19.5.604.

**3**. Sulter G, Steen C, De Keyser J. Use of the Barthel index and modified Rankin scale in acute stroke trials. Stroke. 1999;30(8):1538-1541. doi:10.1161/01.str.30.8.1538.

**4**. Bruno A, Akinwuntan AE, Lin C, et al. Simplified modified rankin scale questionnaire: reproducibility over the telephone and validation with quality of life. Stroke. 2011;42(8):2276-2279. doi:10.1161/STROKEAHA.111.613273.

**5**. The Brain Trauma Foundation. The American Association of Neurological Surgeons. The Joint Section on Neurotrauma and Critical Care. Computed tomography scan features. J Neurotrauma. 2000;17(6-7):597-627. doi:10.1089/neu.2000.17.597.

**6**. Morgenstern LB, Demchuk AM, Kim DH, Frankowski RF, Grotta JC. Rebleeding leads to poor outcome in ultra-early craniotomy for intracerebral hemorrhage. Neurology. 2001;56(10):1294-1299. doi:10.1212/wnl.56.10.1294.

**7**. Shen Z, Wang L, Wu G, Li Q, Ren S, Mao Y. Computed Tomographic Black Hole Sign Predicts Postoperative Rehemorrhage in Patients with Spontaneous Intracranial Hemorrhage Following Stereotactic Minimally Invasive Surgery. World Neurosurg. 2018; 120: e153-e160. doi:10.1016/j.wneu.2018.07.256.

**8**. Zheng WJ, Li LM, Zhu YH, et al. Impact of Surgeon Experience on Postoperative Rehemorrhage in Spontaneous Basal Ganglia Intracerebral Hemorrhage. World Neurosurg. 2019;131: e402-e407. doi:10.1016/j.wneu.2019.07.182.

**9**. Yagi K, Tao Y, Hara K, et al. Does Noncontrast Computed Tomography Scan Predict Rebleeding After Endoscopic Surgery for Spontaneous Intracerebral Hemorrhage? World Neurosurg. 2019;127: e965-e971. doi:10.1016/j.wneu.2019.04.010.

**Supplemental Figure Legend**

**Figure S1:** Flowchart of the patient selection process.

A total of 7091 patients with spontaneous intracerebral hemorrhage were screened; 6780 patients were excluded, and 311 patients were included in the final analysis.

**Supplemental Tables**

**Table S1** Univariate analysis of one-year mRS score (ordinal variable) on categorical variables

| Categorical variables | One-year mRS score, median (IQR) | Z value | *P* value |
| --- | --- | --- | --- |
| Sex |  | -2.23 | 0.03 |
| Male | 3.00(3.00-5.00) |  |  |
| Female | 4.00(3.00-5.00) |  |  |
| Smoking | 3.00(2.00-4.25) | -1.94 | 0.053 |
| Drinking | 3.00(2.00-4.75) | -1.33 | 0.18 |
| Hypertension | 4.00(3.00-5.00) | -2.64 | 0.01 |
| Diabetes | 5.00(3.00-6.00) | -3.01 | 0.003 |
| Antiplatelet | 4.00(2.00-5.50) | -0.43 | 0.67 |
| Arousal level |  | -2.73 | 0.01 |
| Good^a^ | 3.00(3.00-4.00) |  |  |
| Bad^b^ | 4.00(3.00-5.00) |  |  |
| Surgical method |  | -0.91 | 0.37 |
| Microscope | 4.00(3.00-5.00) |  |  |
| Endoscope | 3.00(3.00-5.00) |  |  |
| Surgical approach |  | -4.85 | <0.001 |
| Cortex | 4.00(3.00-5.00) |  |  |
| Sylvian fissure | 3.00(2.00-4.00) |  |  |
| Hematoma location |  | -1.23 | 0.22 |
| Right | 3.00(3.00-5.00) |  |  |
| Left | 3.00(3.00-5.00) |  |  |
| Intraventricular hematoma | 4.00(3.00-5.00) | -3.56 | <0.001 |
| Rebleeding | 5.00(3.00-6.00) | -3.22 | 0.001 |

Abbreviations: mRS, modified Rankin scale; IQR, interquartile range.

a: arousal level = 1, 2 ,3.

b: arousal level = 4, 5.

**Table S2** Univariate analysis of one-year mRS score (ordinal variable) on continuous variables

| Continuous variables | One-year mRS score | | | | | | H value | *P* value |
| --- | --- | --- | --- | --- | --- | --- | --- | --- |
|  | 1 | 2 | 3 | 4 | 5 | 6 |  |  |
| Age, median (IQR), y | 47.50(42.50-51.75) | 50.00(39.25-55.75) | 53.00(46.00-60.00) | 57.00(50.00-69.00) | 61.50(55.00-69.00) | 69.00(59.00-76.25) | 77.89 | <0.001 |
| SBP, median (IQR), mmHg | 166.00(154.00-190.50) | 174.00(161.00-186.00) | 175.00(160.00-190.00) | 177.00(159.00-193.25) | 179.50(160.25-192.25) | 186.00(163.75-204.00) | 6.28 | 0.28 |
| DBP, median (IQR), mmHg | 109.00(90.25-116.25) | 104.00(95.00-120.50) | 104.00(95.00-118.00) | 103.50(92.25-113.00) | 95.50(89.75-106.00) | 99.00(90.00-112.00) | 10.51 | 0.06 |
| GCS score, median (IQR) | 12.00(9.00-12.25) | 10.00(8.00-12.00) | 10.00(8.00-11.00) | 9.00(8.00-10.75) | 9.00(8.00-11.00) | 9.00(7.75-10.00) | 16.40 | 0.01 |
| Hematoma volume, median (IQR), ml | 24.71(20.05-34.58) | 29.50(23.75-36.47) | 36.72(28.69-45.40) | 35.07(28.71-45.13) | 41.41(30.87-51.42) | 43.18(30.31-53.35) | 35.56 | <0.001 |
| Midline shift, median (IQR), mm | 3.30(2.30-5.30) | 4.80(3.60-6.60) | 5.70(4.36-7.40) | 5.55(4.25-7.39) | 6.52(4.77-8.55) | 7.15(4.58-9.25) | 26.35 | <0.001 |
| Surgical timing, median (IQR), min | 407.50(351.50-573.75) | 503.00(312.25-902.50 | 504.00(348.00-837.50) | 352.50(281.00-643.75) | 490.50(326.25-657.50) | 399.50(316.75-697.25) | 10.19 | 0.07 |

Abbreviations: mRS, modified Rankin scale; IQR, interquartile range; SBP, systolic blood pressure; DBP, diastolic blood pressure; GCS, Glasgow Coma Scale.

**Table S3** Univariate analysis of one-year mRS score (dichotomous variable)

| Variables | One-year mRS score, n (%) | | χ2/Z | *P* value |
| --- | --- | --- | --- | --- |
|  | Good^a^ | Bad^b^ |  |  |
| Sex |  |  | 8.08 | 0.004 |
| Male | 128(76.65) | 89(61.81) |  |  |
| Female | 39(23.35) | 55(38.19) |  |  |
| Smoking | 57(34.13) | 33(22.92) | 4.73 | 0.03 |
| Drinking | 56(33.53) | 40(27.78) | 1.20 | 0.27 |
| Hypertension | 94(56.29) | 98(68.06) | 4.53 | 0.03 |
| Diabetes | 7(4.19) | 12(8.33) | 2.31 | 0.13 |
| Antiplatelet | 2(1.20) | 3(2.08) | NA | 0.67 |
| Arousal level |  |  | 7.62 | 0.01 |
| Good^c^ | 141(84.43) | 103(71.53) |  |  |
| Bad^d^ | 26(15.57) | 41(28.47) |  |  |
| Surgical method |  |  | 13.24 | <0.001 |
| Microscope | 77(46.11) | 96(66.67) |  |  |
| Endoscope | 90(53.89) | 48(33.33) |  |  |
| Surgical approach |  |  | 11.98 | 0.001 |
| Cortex | 124(74.25) | 129(89.58) |  |  |
| Sylvian fissure | 43(25.75) | 15(10.42) |  |  |
| Hematoma location |  |  | 1.77 | 0.18 |
| Right | 81(48.50) | 59(40.97) |  |  |
| Left | 86(51.50) | 85(59.03) |  |  |
| Intraventricular hematoma | 57(34.13) | 69(47.92) | 6.10 | 0.01 |
| Rebleeding | 11(6.59) | 19(13.19) | 3.87 | 0.049 |
| Age, median (IQR), y | 51.00(44.00-57.00) | 64.50(53.25-72.00) | -8.09 | <0.001 |
| SBP, median (IQR), mmHg | 174.00(160.00-188.00) | 180.00(162.00-193.75) | -1.64 | 0.10 |
| DBP, median (IQR), mmHg | 105.00(95.00-119.00) | 99.00(90.25-112.00) | -2.89 | 0.004 |
| GCS score, median (IQR) | 10.00(8.00-12.00) | 9.00(8.00-10.00) | -3.24 | 0.01 |
| Hematoma volume, median (IQR), ml | 32.59(26.23-40.48) | 39.37(29.93-51.00) | -3.66 | <0.001 |
| Midline shift, median (IQR), mm | 5.40(3.90-7.00) | 6.39(4.52-8.16) | -3.26 | 0.01 |
| Surgical timing, median (IQR), min | 490.00(345.00-864.00) | 410.00(315.00-665.75) | -2.64 | 0.01 |

Abbreviations: mRS, modified Rankin scale; IQR, interquartile range; SBP, systolic blood pressure; DBP, diastolic blood pressure; GCS, Glasgow Coma Scale; NA, not applicable.

a: mRS score = 1, 2, 3.

b: mRS score = 4, 5, 6.

c: arousal level = 1, 2, 3.

d: arousal level = 4, 5.

**Table S4** Univariate analysis of rebleeding

| Variables | Rebleeding, n (%) | | χ2/Z | *P* value |
| --- | --- | --- | --- | --- |
|  | Yes | No |  |  |
| Sex |  |  | 1.65 | 0.20 |
| Male | 24(80.00) | 193(68.68) |  |  |
| Female | 6(20.00) | 88(31.32) |  |  |
| Smoking | 7(23.33) | 83(29.54) | 0.51 | 0.48 |
| Drinking | 7(23.33) | 89(31.67) | 0.88 | 0.35 |
| Hypertension | 23(76.67) | 169(60.14) | 3.13 | 0.08 |
| Diabetes | 5(16.67) | 14(4.98) | NA | 0.03 |
| Antiplatelet | 1(3.33) | 4(1.42) | NA | 0.40 |
| Arousal level |  |  | 0.05 | 0.83 |
| Good^a^ | 24(80.00) | 220(78.29) |  |  |
| Bad^b^ | 6(20.00) | 61(21.71) |  |  |
| Surgical method |  |  | 8.83 | 0.003 |
| Microscope | 9(30.00) | 164(58.36) |  |  |
| Endoscope | 21(70.00) | 117(41.64) |  |  |
| Surgical approach |  |  | 1.64 | 0.20 |
| Cortex | 27(90.00) | 226(80.43) |  |  |
| Sylvian fissure | 3(10.00) | 55(19.57) |  |  |
| Hematoma location |  |  | 0.04 | 0.85 |
| Right | 14(46.67) | 126(44.84) |  |  |
| Left | 16(53.33) | 155(55.16) |  |  |
| Intraventricular hematoma | 17(56.67) | 109(38.79) | 3.59 | 0.06 |
| Age, median (IQR), y | 54.50(45.75-64.50) | 55.00(48.00-66.00) | -0.69 | 0.49 |
| SBP, median (IQR), mmHg | 183.00(164.00-193.50) | 176.00(160.00-191.00) | -1.25 | 0.21 |
| DBP, median (IQR), mmHg | 107.00(96.75-118.50) | 101.00(93.00-114.50) | -1.13 | 0.26 |
| GCS score, median (IQR) | 9.00(8.00-11.25) | 9.00(8.00-11.50) | -0.20 | 0.84 |
| Hematoma volume, median (IQR), ml | 43.19(29.60-59.51) | 34.51(26.85-44.96) | -2.41 | 0.02 |
| Midline shift, median (IQR), mm | 6.08(4.10-8.54) | 5.70(4.20-7.45) | -1.04 | 0.30 |
| Surgical timing, median (IQR), min | 403.00(333.25-776.50) | 460.00(317.00-730.50) | -0.08 | 0.93 |

Abbreviations: IQR, interquartile range; SBP, systolic blood pressure; DBP, diastolic blood pressure; GCS, Glasgow Coma Scale; NA, not applicable.

a: arousal level = 1, 2, 3.

b: arousal level = 4, 5.

**Table S5** Univariate analysis of three-month mortality

| Variables | Three-month mortality, n (%) | | χ2/Z | *P* value |
| --- | --- | --- | --- | --- |
|  | Yes | No |  |  |
| Sex |  |  | 0.20 | 0.65 |
| Male | 17(73.91) | 200(69.44) |  |  |
| Female | 6(26.09) | 88(30.56) |  |  |
| Smoking | 6(26.09) | 84(29.17) | 0.10 | 0.75 |
| Drinking | 6(26.09) | 90(31.25) | 0.27 | 0.61 |
| Hypertension | 17(73.91) | 175(60.76) | 1.56 | 0.21 |
| Diabetes | 3(13.04) | 16(5.56) | NA | 0.16 |
| Antiplatelet | 1(4.35) | 4(1.39) | NA | 0.32 |
| Arousal level |  |  | NA | >0.999 |
| Good^a^ | 18(78.26) | 226(78.47) |  |  |
| Bad^b^ | 5(21.74) | 62(21.53) |  |  |
| Surgical method |  |  | 0.28 | 0.60 |
| Microscope | 14(60.87) | 159(55.21) |  |  |
| Endoscope | 9(39.13) | 129(44.79) |  |  |
| Surgical approach |  |  | NA | 0.59 |
| Cortex | 20(86.96) | 233(80.90) |  |  |
| Sylvian fissure | 3(13.04) | 55(19.10) |  |  |
| Hematoma location |  |  | 2.13 | 0.14 |
| Right | 7(30.43) | 133(46.18) |  |  |
| Left | 16(69.57) | 155(53.82) |  |  |
| Intraventricular hematoma | 15(65.22) | 111(38.54) | 6.29 | 0.01 |
| Rebleeding | 6(26.09) | 24(8.33) | NA | 0.02 |
| Age, median (IQR), y | 66.00(51.00-65.00) | 55.00(47.25-65.00) | -2.57 | 0.01 |
| SBP, median (IQR), mmHg | 178.00(161.00-190.00) | 176.50(160.00-191.00) | -0.08 | 0.93 |
| DBP, median (IQR), mmHg | 98.00(88.00-118.00) | 102.50(94.00-115.00) | -0.59 | 0.55 |
| GCS score, median (IQR) | 9.00(8.00-10.00) | 9.00(8.00-12.00) | -0.64 | 0.53 |
| Hematoma volume, median (IQR), ml | 44.73(23.31-51.23) | 34.99(27.51-45.15) | -0.46 | 0.64 |
| Midline shift, median (IQR), mm | 6.30(4.80-7.98) | 5.65(4.16-7.58) | -0.89 | 0.38 |
| Surgical timing, median (IQR), min | 339.00(310.00-495.00) | 466.50(328.50-753.00) | -1.84 | 0.67 |

Abbreviations: IQR, interquartile range; SBP, systolic blood pressure; DBP, diastolic blood pressure; GCS, Glasgow Coma Scale; NA, not applicable.

a: arousal level = 1, 2, 3.

b: arousal level = 4, 5.

**Table S6** Multivariable logistic regression of rebleeding. The cortex approach was the control group, and the Sylvian fissure approach was the treatment group.

| Variables | Odds Ratio (95% CI) | *P* value | VIF |
| --- | --- | --- | --- |
| Diabetes | 3.32(1.06-10.35) | 0.04 | 1.01 |
| Surgical method | 2.67(1.05-6.80) | 0.04 | 1.24 |
| Hematoma volume, ml | 1.02(1.00-1.04) | 0.047 | 1.12 |
| Surgical approach | 1.11(0.27-4.56) | 0.88 | 1.21 |

Abbreviation: CI, confidence interval; VIF, variance inflation factor.

**Table S7** Multivariable logistic regression of three-month mortality. The cortex approach was the control group, and the Sylvian fissure approach was the treatment group.

| Variables | Odds Ratio (95% CI) | *P* value | VIF |
| --- | --- | --- | --- |
| Intraventricular hematoma | 2.45(0.97-6.22) | 0.06 | 1.03 |
| Rebleeding | 3.55(1.19-10.58) | 0.02 | 1.02 |
| Age, y | 1.05(1.01-1.09) | 0.01 | 1.01 |
| Surgical approach | 0.85(0.23-3.18) | 0.81 | 1.01 |

Abbreviation: CI, confidence interval; VIF, variance inflation factor.

**Table S8** Univariate analysis before propensity score matching

| Variables | Surgical approach, n (%) | | χ2/Z value | *P* value |
| --- | --- | --- | --- | --- |
|  | Cortex | Sylvian fissure |  |  |
| Sex |  |  | 0.61 | 0.43 |
| Male | 179(70.75) | 38(65.52) |  |  |
| Female | 74(29.25) | 20(34.48) |  |  |
| Smoking | 68(26.88) | 22(37.93) | 2.80 | 0.09 |
| Drinking | 82(32.41) | 14(24.14) | 1.51 | 0.22 |
| Hypertension | 163(64.43) | 29(50.00) | 4.16 | 0.04 |
| Diabetes | 16(6.32) | 3(5.17) | NA | >0.999 |
| Antiplatelet | 5(1.98) | 0(0) | NA | 0.59 |
| Arousal level |  |  | 0.28 | 0.59 |
| Good^a^ | 200(79.05) | 44(75.86) |  |  |
| Bad^b^ | 53(20.95) | 14(24.14) |  |  |
| Surgical method |  |  | 48.38 | <0.001 |
| Microscope | 117(46.25) | 56(96.55) |  |  |
| Endoscope | 136(53.75) | 2(3.45) |  |  |
| Hematoma location |  |  | 0.38 | 0.54 |
| Right | 116(45.85) | 24(41.38) |  |  |
| Left | 137(54.15) | 34(58.62) |  |  |
| Intraventricular hematoma | 108(42.69) | 18(31.03) | 2.66 | 0.10 |
| Rebleeding | 27(10.67) | 3(5.17) | 1.64 | 0.20 |
| Age, median (IQR), y | 56.00(47.50-66.50) | 53.00(48.00-65.00) | -0.62 | 0.54 |
| SBP, median (IQR), mmHg | 178.00(161.50-192.00) | 170.50(153.00-187.75) | -1.64 | 0.10 |
| DBP, median (IQR), mmHg | 101.00(94.00-114.00) | 109.50(90.00-118.25) | -1.16 | 0.25 |
| GCS score, median (IQR) | 9.00(8.00-11.00) | 10.00(8.75-12.00) | -1.61 | 0.11 |
| Hematoma volume, median (IQR), ml | 36.97(28.08-49.25) | 30.05(21.94-36.33) | -4.46 | <0.001 |
| Midline shift, median (IQR), mm | 6.00(4.50-7.86) | 4.25(2.98-6.00) | -4.42 | <0.001 |
| Surgical timing, min | 480.00(329.00-754.00) | 402.50(300.00-582.75) | -1.39 | 0.17 |

Abbreviations: NA, not applicable; IQR, interquartile range; SBP, systolic blood pressure; DBP, diastolic blood pressure; GCS, Glasgow Coma Scale.

a: arousal level = 1, 2, 3.

b: arousal level = 4, 5.

**Table S9** Propensity score-adjusted multivariable logistical regression for one-year mRS score (dichotomous variable). The cortex approach was the control group, and the Sylvian fissure approach was the treatment group.

| Variables | Odds Ratio (95% CI) | *P* value | VIF |
| --- | --- | --- | --- |
| Surgical method | 15.65(5.84-42.00) | <.001 | 2.41 |
| Surgical approach | 5.83(2.38-14.27) | <.001 | 1.39 |
| Rebleeding | 0.19(0.07-0.51) | 0.001 | 1.05 |
| Age, y | 1.91(0.88-0.93) | <.001 | 1.11 |
| GCS score | 1.29(1.12-1.50) | 0.001 | 1.05 |
| Hematoma volume, ml | 0.98(0.96-1.00) | 0.045 | 1.39 |

Abbreviations: mRS, modified Rankin scale; CI, confidence interval; VIF, variance inflation factor; GCS, Glasgow Coma Scale.

**Table S10** Propensity score-adjusted multivariable logistical regression for rebleeding. The cortex approach was the control group, and the Sylvian fissure approach was the treatment group.

| Variables | Odds Ratio (95% CI) | *P* value | VIF |
| --- | --- | --- | --- |
| Diabetes | 3.32(1.06-10.42) | 0.04 | 1.01 |
| Surgical method | 2.70(0.72-10.18) | 0.14 | 2.29 |
| Hematoma volume, ml | 1.02(1.00-1.04) | 0.06 | 1.30 |
| Surgical approach | 1.10(0.24-5.06) | 0.90 | 1.39 |

Abbreviation: CI, confidence interval; VIF, variance inflation factor.

**Table S11** Propensity score-adjusted multivariable logistical regression for three-month mortality. The cortex approach was the control group, and the Sylvian fissure approach was the treatment group.

| Variables | Odds Ratio (95% CI) | *P* value | VIF |
| --- | --- | --- | --- |
| Rebleeding | 4.56(1.53-13.58) | 0.01 | 1.02 |
| Age, y | 1.06(1.02-1.10) | 0.01 | 1.01 |
| Surgical approach | 0.60(0.14-2.54) | 0.48 | 1.39 |

Abbreviation: CI, confidence interval; VIF, variance inflation factor.
